# PDBrestore: A Free Web Interface for Processing and Fixing Protein Chains From Raw PDB Files

**DOI:** 10.1002/jcc.70124

**Published:** 2025-05-14

**Authors:** Piero Procacci

**Affiliations:** ^1^ Department of Chemistry University of Florence Sesto Fiorentino Italy

**Keywords:** gap handling, missing residues, molecular dynamics, PDB manipulation, PDB repair

## Abstract

We present PDBrestore, a free web interface for repairing protein PDB chains extracted from either a local PDB file or a PDB file downloaded from the Protein Data Bank. PDBrestore performs several key tasks: It adds hydrogen atoms, completes missing atoms in side chains, fills gaps in the sequence, derives the itp parameter file for a ligand according to the GAFF2 force field for GROMACS applications, and generates a reasonably pre‐equilibrated solvated simulation box. The interface is designed to streamline the cumbersome preparatory work required to set up an initial protein‐ligand coordinates PDB file for use in drug design projects, such as free energy perturbation or thermodynamic integration calculations of ligand binding affinities. Additionally, PDBrestore is available as a command‐line application within the open‐source ORAC distribution, which can be freely downloaded from the website: www1.chim.unifi.it/orac.

## Graphical abstract

PDBrestore is a freely accessible web interface to repair raw PDB files fetched from the Protein Data Bank for molecular dynamics applications. The interface, with minimal end‐user intervention, repairs common PDB defects such as missing atoms, incomplete side chains, missing residues, non‐standard residue names or non‐standard atom labels, manages the presence of cofactors, ligands, or post‐translational modifications such as disulfide bridges, and provides a pre‐equilibrated simulation box including explicit solvent.

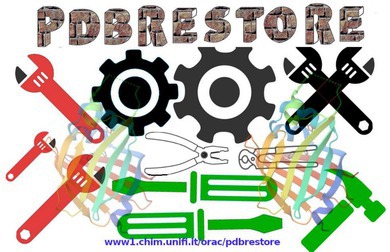

## 1 Introduction

Powered by the remarkable growth in the last decades of computer resources and the flood of data on ligand‐protein properties [1], computational drug design has established itself as an essential tool for drug discovery in industrial as well as academic settings. The consensus machinery in today's computer‐aided drug design relies on a hierarchical, funnel‐shaped computational pipeline, whereby hits from cost‐effective docking or supervised machine learning (ML) approaches on a given protein target are often purged of false positives using expensive molecular dynamics (MD) techniques with a full atomistic description of the system to eventually proceed to wet‐lab validation [2, 3, 4, 5, 6].

MD assessment on streamlined hits from ML or docking exploits techniques such as Free Energy Perturbation [7, 8, 9, 10, 11], Thermodynamic integration [4, 12], Metadynamics [13, 14], or non‐equilibrium simulations [15, 16, 17]. The common starting point for any MD approach to binding affinity calculations is the availability of a reasonable initial atomic structure of the protein target under scrutiny, possibly supplemented with cofactors and ligands. Protein atomic coordinates are usually extracted from X‐ray, cryo‐EM, or NMR data deposited in the Protein Data Bank (PDB). The preparation of the initial MD configuration from a raw PDB file involves several cumbersome steps, related to missing atoms, incomplete side‐chains, missing residues, non‐standard residue names or non‐standard atom labels, presence of cofactors such as metals or prosthetic groups. In general, such tasks can be distressing and error‐prone, subject to diverse inconveniences and shortcomings that make the whole process troublesome and time‐consuming.

Several software tools have been designed to help with this endeavour. The most popular ones include both commercial molecular visualization packages, such as PyMOL by Schroedinger inc [18], Modeller [19], Swiss‐PdbViewer (DeepView) [20], CHARMM‐GUI [21, 22] or free or open source tools such as Chimera [23], pdb‐tools [24], VMD [25], Protein Repair & Analysis Server [26] (PRAS), or PDBfixer [27]. Each of these tools, which invariably require local installation (except for the public web servers PRAS at https://www.protein‐science.com/ and the CHARMM‐GUI interface at charmm‐gui.org) has its strengths and weaknesses. For example, the PRAS web server has limited capabilities (e.g., does not repair internal gaps or incorporate metals or ligands), molecular visualization tools such as PyMol or Chimera, require interaction with a complex graphical interface for structure editing. Others, like PDBfixer or CHARMM‐GUI, may fail in delivering optimally minimized structures in key areas, such as the gap reparations, cysteine handling, metal incorporation, and their coordinating units.

In this study, we present PDBrestore, a free web tool for processing protein chains from PDB files with the aim of delivering refined all‐atom ligand‐protein structures ready to use in MD applications for drug discovery. The PDBrestore web server is freely accessible at the internet address http://www1.chim.unifi.it/orac/pdbrestore and can be used with any browser with no need for registration. The end‐user is required to upload a local PDB file or to specify the four‐character alphanumeric code corresponding to the PDB structure in the Protein Data Bank. PDBrestore first analyzes the PDB file, delivering structural information on protein chains, gap locations, and heteroatoms. End‐user selections are made via a web interface where the protein chain, the ligand, gap repairing, and the generation of an equilibrated MD box with explicit solvent can be optionally check‐marked. Results, if the refinement process is successful, can be uploaded in the form of a compressed tar archive. This archive includes the refined PDB files (with or without water solvent), as well as the itp files [28] of the ligand for GROMACS application. PDBrestore is part of the ORAC [29] distribution and can be run as a command‐line application after ORAC local installation and setup.

The present paper is organized as follows. In the Section “The PDBrestore interface: Underlying Methodology and Work Flow” we describe the basic operating principles of the PDBrestore tool, with details on the side‐chain and the gap repair mechanism, identification of CYS‐CYS disulfide bridges, metals, refinement of coordinating residues, ligand optimization, and minimization process. In the section “Results”, we discuss the performance of PDBrestore on a set of ≃ 20000 protein chains and protein‐ligand systems randomly taken from the 220,000 (as of January 2025) publicly available structures at the PDB ftp site ftp://ftp.ebi.ac.uk. In the conclusion sections, the strengths and limitations of the interface are highlighted, and perspectives for further improvement are envisioned.

## 2 The PDBrestore Interface: Underlying Methodology and Work Flow

**FIGURE 1:**
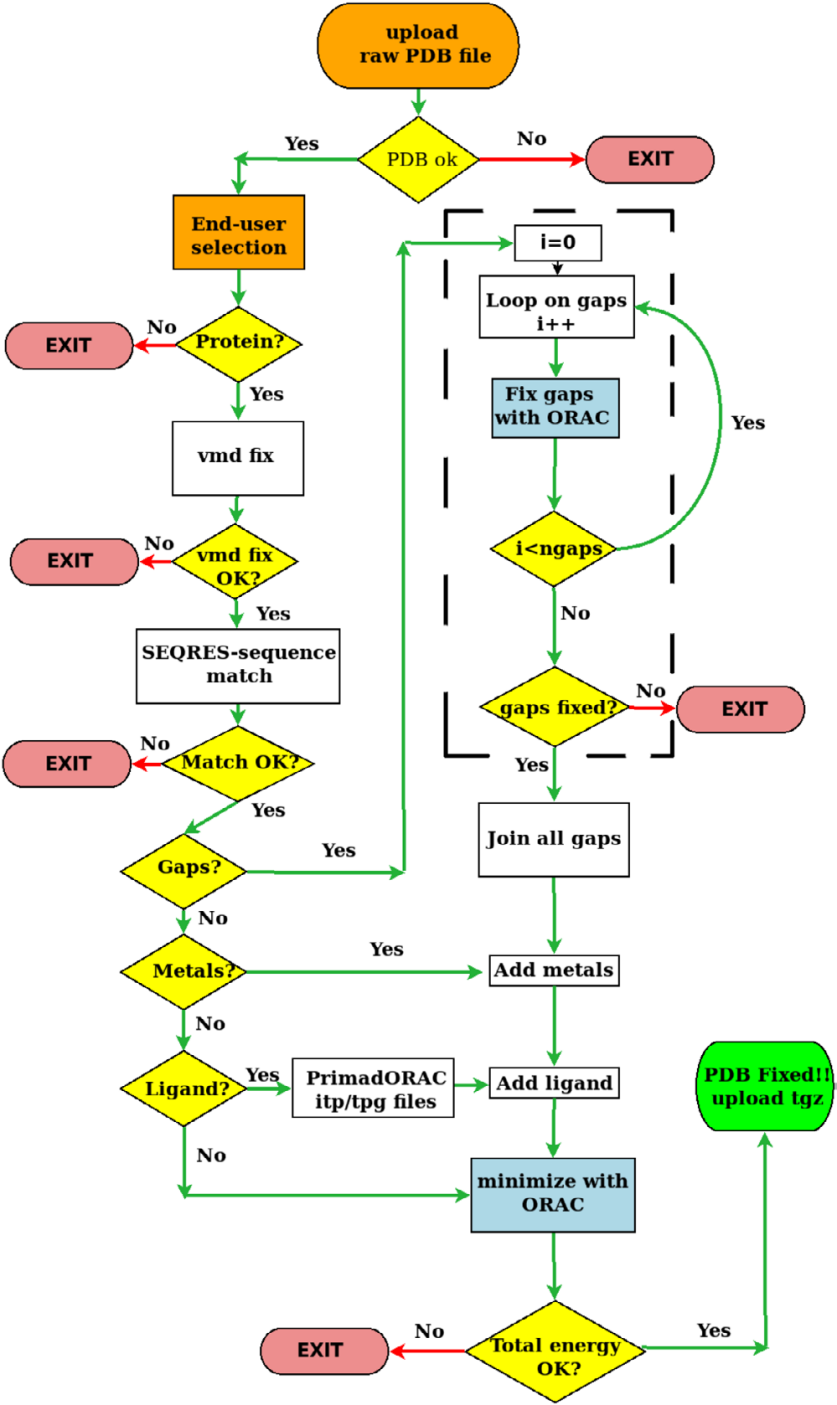
Flowchart of the PBDrestore interface.

PDBrestore is focused on providing a reasonable starting point for the structure of a *single* protein chain and ligand for MD applications. This includes the incorporation of cofactors such as metals, the identification of disulfide bridges, the delivery of ligand force field parameters, and the addition and optimization of missing residues or side chains. The flowchart of the PDBrestore web interface is provided in Figure 1.

The raw PDB files can be either uploaded from a local copy or fetched from the ftp public PDB interface. Upon PDB uploading, the interface provides succinct structural information found in the raw structure. The end‐user is directed to a page where he/she can make his/her choices regarding the chain and ligand to be included in the optimized target structure. If the end‐user selects a protein chain, the chain is extracted from the raw PDB file and fed to the VMD‐PSF plugin [25] that adds the missing atoms in side chains, if any. Gaps, if present, are not fixed at this stage. The VMD‐PSF the plugin produces a final protein chain structure by adding all hydrogen atoms with histidine in the ϵ tautomeric form and with all protonated cysteines regardless of the potential presence of S‐S disulfide bridges or of the actual protonation state in the case of sulfur metal coordination.

The interface proceeds by matching the amino acid sequence from the SEQRES records of the selected chain to the sequence perceived from the backbone atoms provided in the ATOM records. If gaps are found, each gap in the selected protein chain is processed individually using a divide and conquer strategy. Simultaneous generation and optimization of all gaps would, in fact, require the identification of the optimal mutual orientations of all the missing sequences before closing the gaps, possibly producing inter‐gap interference during the minimization and failure of the optimization process.

The single‐gap repair module of the PDBrestore tool is highlighted in the central dashed box in Figure 1. The missing acidic amino subsequence is generated by the ALLTRANS ancillary tool provided in the ORAC distribution. The N‐terminal of the ALLTRANS‐generated gap sub‐sequence is then joined to the first residue before the gap in the protein chain, examining its CA and C coordinates. The chain‐joined all‐trans missing sequence is then oriented along six directions with its C‐terminal residue pointing to the vertices of an octahedron. For each orientation, the C‐terminal of the gap sub‐sequence is then connected via ORAC minimization to the N‐terminal of the residue in the chain sequence that delimits the gap upward. This stage produces the coordinates of six differently oriented subsequences of the closed gaps, including the first and last downward and upward residues delimiting the gap. In this preliminary process of subsequent closure, the coordinates of the two chain residues delimiting the gap are kept fixed to the original coordinates in the chain. The resulting atomic coordinates of the joined sub‐sequences are then inserted into the main chain coordinates, filling the gap. A corresponding ORAC minimization input file is produced by correcting the topology of protonated cysteines into cysteines connected via a disulfide bridge (if any).

Each tentative gap orientation is initially tested for steric clashes with the protein chain using the ORAC minimization module. The optimal orientation then undergoes a fast conjugate gradient minimization to produce the optimized structure of the chain with the subsequence included. During this gap‐fixing process, no restraints are imposed on the chain or subsequent atomic coordinates, allowing them to adjust freely for optimal results.

As the operation proceeds, the interface produces an output indicating the outcome of the various tested gap orientations, displaying the total energy of the protein (in kJ/mol) with the gap subsequence included. A negative energy of approximately −100 kJ/mol per amino acid is indicative of a successful minimization stage.

Once all gaps are processed one by one in the gap‐repairing module, PDBrestore proceeds to the final stage. This stage involves inserting the coordinates of all the repaired gap subsequences into the VMD‐generated protein chain PDB file, incorporating metals, potentially switching to the δ histidine based on the N‐metal coordinating distance, and inserting the user‐selected ligand. The ORAC input for the final minimization process includes the correct topological specification for the cysteines and the tautomers of the coordinating histidines (when metals such as ZN or CU are present). The force field used in the minimization stage is the AMBER99SB‐ILDN parameter set [30]. The topology and parameter (TPG/PRM) set for the ligand are generated by the PrimaDORAC tool [31] according to the GAFF2 force field [32, 33]. In case of prosthetic groups coordinating a metal such as heme or bacteriochlorophyll, PrimaDORAC generates the TPG/PRM set of the molecule in the correct protonation state, excluding the metal. The TGP/PRM of metallic ions is treated using the nonbonded force fields developed by Merz and coworkers [34] (TIP3P [35] variant).

In the final stage, depending on the end‐user's initial choice, a cubic equilibrated MD simulation box including the protein‐ligand system and explicit water molecules is eventually produced using ORAC. Equilibration is conducted in the isothermal‐isobaric ensemble at T=300 K and P = 1 atm up to stationarity of the sidelength of the cubic MD box using a Parrinello‐Rahman extended Lagrangian [36] with isostress scaling [37] and a series of Nosé thermostats [38] for pressure and temperature control, respectively. The water is described by the OPC3 force field [39].

If everything goes as planned, the interface provides a hyperlink for downloading a compressed tar archive. This archive includes the repaired PDB file of the selected chain, the itp file of the ligand for GROMACS applications and, optionally, the equilibrated coordinates of the solvated system in a cubic MD box.

## 3 Results

### 3.1 Statistics on the PBD

**FIGURE 2:**
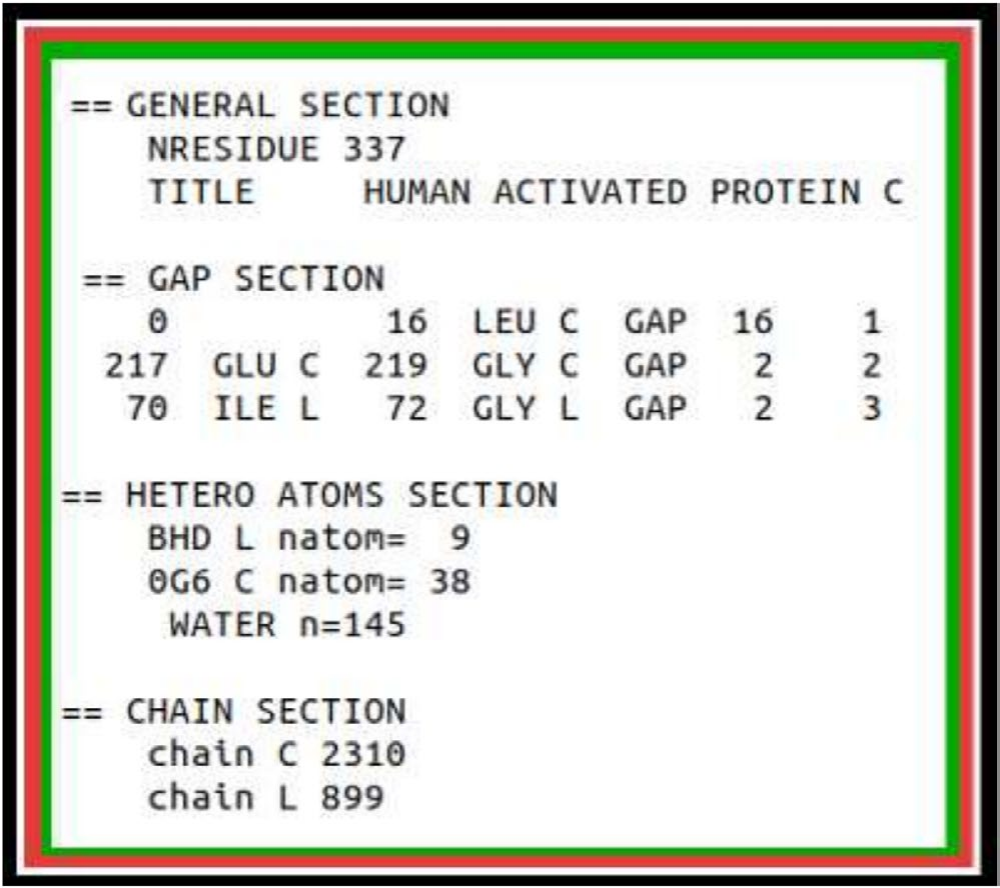
Example of a PDBrestore structural information output obtained by processing the PDB file 1AUT.

The PBD contains, as of January 2025, approximately 220,000 entries. In Figure 2, we show a typical output produced by the PDBrestore interface with structural information on the target PDB entry (1AUT). The PDB structure 1AUT includes a total of 338 residues, distributed into two protein chains with 2310 and 899 atoms (C and L). Both chains contain one *internal* gap missing one residue. The protein complex 1AUT further incorporates two ligands and 415 water molecules.

**FIGURE 3:**
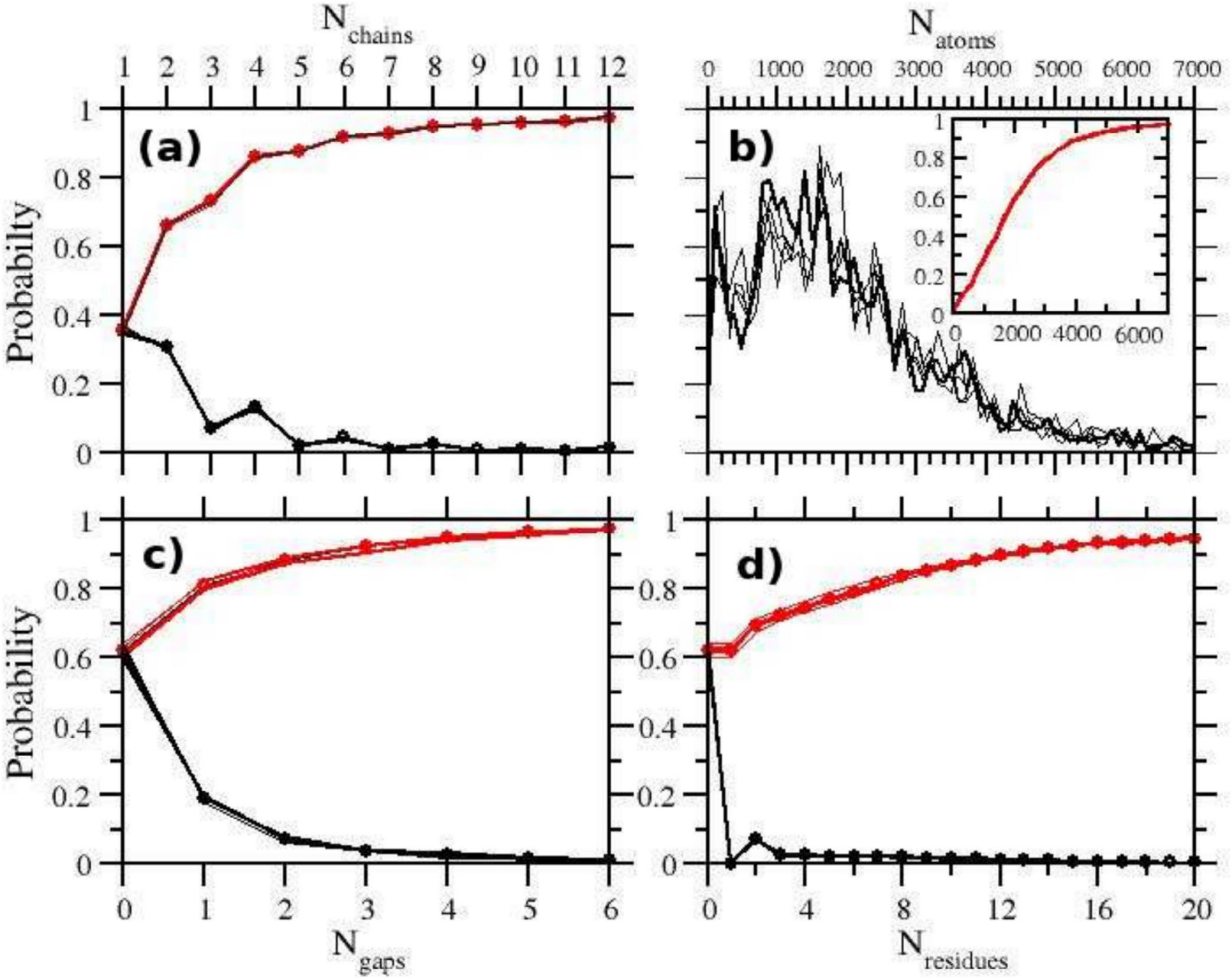
Statistics on the full PDB database as obtained by randomly sampling 8,000 entries from the ftp site ftp://ftp.ebi.ac.uk/pub/databases as of January 2025: (a) distribution of number of chains in PDB files. In black and red colors, the distribution and cumulative probability are shown, respectively. The thick lines and circles refer to the global average. The thin lines are the results obtained from four independent samples, each with 2,000 PDB entries. (a) number of chains; (b) chain length (in heavy atoms); (c) and (d) gaps found in the protein chains (c) distribution of the average gap length in the protein chains.

The PDB database has a highly diverse content, from entries with a single protein chain to structures with more than 20 protein chains. Multiple protein chains may refer to symmetry‐related units in the crystallographic cell, dimers or oligomers, or a complex of proteins of different nature and function. Non‐protein elements may include nucleic acids, small molecules (e.g., drugs, inhibitors, substrates) ATP (adenosine triphosphate), GTP (guanosine triphosphate), GDP (guanosine diphosphate), metal ions, and water. In Figure 3, we show a succinct statistical overview of the PDB, obtained by sampling randomly about 8,000 PDB entries from the public database. The reported analysis focuses solely on protein chains. DNA or RNA chains are not currently supported by PDBrestore. Uncertainty on the computed distributions has been assessed by using the data of four samples, each containing 2,000 PDB entries.

The distribution of the number of protein chains per PDB entry in the database (Figure 3a) shows a decreasing behavior with nearly 40% of the entries including a single protein chain. Interestingly, the probability distribution of the number of chains exhibits a superimposed oscillatory behavior up to $N_{\text{chains}}=8$, where the probability of an odd number of chains is consistently lower than that of the following even number. This trend, observed in each of the four 2,000 entries, independent sample (see Figure 3 caption), likely reflects the fact that most of the PDB entries are X‐ray structures derived from crystallographic cells containing an even number of symmetry‐ equivalent units. According to the cumulative probability distribution (red curve), less than 15% of the PDB entries contain more than four protein chains, and less than 2% more than 15 chains.

The chain length distribution probability (shown in Figure 3b as a function of the number of heavy atoms) exhibits a maximum at approximately 1,300–1,500 (corresponding to 150–170 residues), with discernible differences observed among the independent samples. Nonetheless, the cumulative probability distribution of the chain length is similar for the four independent samples, showing that 75% of the protein chains have fewer than 3,000 heavy atoms (≃ 350 residues), and only 2% are large chains with more than 8,000 heavy atoms.

Finally, in Figure 3c,d, we report the statistics on the presence of internal gaps in the PDB entries. While many of the protein chains are missing parts of the N‐ and/or C‐terminus, due to the disordered nature of these structural elements, *internal* gaps should be less frequently observed in X‐ray data. Remarkably, nearly 40% of the protein chains exhibit at least one internal gap, with a mean gap length of 17±1 residues. If we exclude from the PDB samples the outliers with gap length greater than 100, the average gap length drops to 10±0.5 residues. Gap location generally involves loops or regions that lack secondary structure [40]. The presence of gaps of 10 or more amino acids represents a significant challenge for setting up of a starting configuration of the system for MD applications.

### 3.2 PDBrestore Performances

PDBrestore performance was assessed by running the command line variant provided in the ORAC distribution on three independent samples, each consisting of 7232, 7695, and 7393 protein chains randomly extracted from the PDB. Results are reported in Table 1.

**TABLE 1:** Success and failure rates of the PDBrestore interface on three independent samples of protein chains randomly extracted from the PDB.

	All						
	Success				Failure		
	$N_{\text{chains}}$	OK	$N_{\text{chains}}^{\mathrm{with}\;\mathrm{gaps}}$	OK	Seq.	Gap	Other
1	7,232	5,986 (82.8%)	2,671	2,107 (78.9%)	242 (3.3%)	564 (7.8%)	440 (6.1%)
2	7,695	6,605 (85.8%)	2,798	2,282 (81.6%)	224 (2.9%)	516 (6.7%)	350 (4.5%)
3	7,393	6,334 (85.7%)	3,954	3,268 (82.7%)	373 (5.0%)	485 (6.5%)	201 (2.7%)
**With ligand**							
1	2,192	1,643 (75.0%)	801	697 (87.0%)	5 (<0.1%)	104 (4.7%)	440 (18.4%)
2	2,329	1,952 (83.8%)	815	788 (96.7%)	3 (<0.1%)	24 (1.1%)	350 (15.5%)
3	1,439	1,285 (89.3%)	744	617 (82.9%)	7 (1.9%)	60 (2.8%)	87 (6.0%)

*Note:* The first three rows refer to the overall statistics. The last three rows refer to the subset of the samples including a ligand.

The average success rate of the PDBrestore tool is 83.8±1.4% with minor variations observed across the three independent PDB samples, demonstrating the robustness of the test. The success rate is slightly degraded for the subset of protein chains exhibiting at least one internal gap, amounting to 81.1 ±1.5. Failures (≃16%) are primarily due to:

- non‐fullfillment of sequence matching,
- inability of the ORAC minimization to fix one gap,
- miscellaneous issues such as VMD fix failures,
- chains with only alpha traces,
- presence of unknown, unconventional, or non‐standard amino acids,
- failure of the minimization process to join multiple internal gaps,
- or presence of defective ligands.

For the subset of protein chains including a ligand (metals and solvent molecules are excluded as ligands), the success rate is similar (82.7±5,8) to the overall rate, with most of the failures due to generic defective PDB coordinates rather than inability to fix the gap or to sequence discrepancies.

One important feature of the PDBrestore interface is the delivery, at the end‐user's discretion, of an MD box of appropriate size including the repaired ligand‐receptor system and explicit solvent molecules. The solvated MD box is cubic, and its initial size is determined by first reorienting the ligand‐receptor coordinates according to the principal axis frame. The side length is set such that the minimum distance between any atom of the protein‐ligand complex and the box walls is at least 15 Å. Water molecules at the standard density are then generated with random orientations, and any water molecules overlapping with the ligand‐receptor system are removed.

**FIGURE 4:**
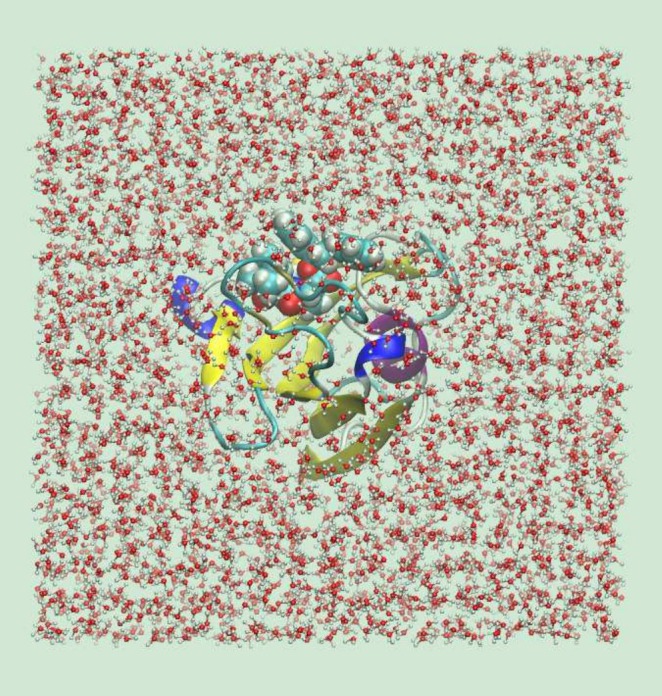
Solvated MD box (VMD orthographic view) delivered by the PDBrestore web interface for an FKBP12‐ligand complex (PDB file 1FKG). The protein, ligand, and water are shown as new cartoons, Van der Waals spheres, and ball and stick representations, respectively.

PDBrestore then performs a short MD simulation at constant pressure and temperature (a few tens of ps) aimed at reshuffling the water molecules according to the force field and adjusting the box size to the standard thermodynamic conditions. The success rate of the solvation stage, by design, is close to 100%. The failure of PDBrestore to produce the solvated box is due to prior failures in repairing the raw PDB file. In Figure 4, we show an example of solvated MD box produced by the PDBrestore interface.

Finally, it is important to note that the current version of the PDBrestore web application has certain limitations when processing protein chains and delivering solvated boxes. These limitations are related to the total number of atoms in the system. Specifically, chains with more than 9,000 heavy atoms are not processed by the interface. Consequently, solvated boxes with more than approximately 120,000 atoms are generally not delivered. However, the 9,000‐atom threshold for chain size is quite generous, as approximately 97% of the protein chains in the PDB have fewer heavy atoms than this threshold, as can be inferred from Figure 3b. These limitations in the use of PDBrestore are due to the current hardware on which the web server is running, as explained in more detail in the Conclusion section.

### 3.3 PDBrestore Versus PDBfixer and CHARMM‐Gui

In this section, we examine in detail the final output produced by the PDBrestore, PDBfixer, and CHARMM‐GUI applications on two challenging PDB structures downloaded from the public PDB ftp site, namely 7NNG and 6A4I. While available PDB repairing web servers such as PRAS [26] or pdb‐tools [24], have limited capabilities, PDBfixer, and CHARMM‐GUI are probably the most popular and a successful application among the many tools for repairing defective PDB files. PDBFixer, an ancillary software of the OpenMM molecular dynamics program [27], generates its interface through a local web browser, functioning as a single‐user desktop application. Like PDBrestore, PDBfixer optimizes missing atoms, side chains, ligands, metals, or entire missing loops through its easy‐to‐use browser interface with minor end‐user intervention. Like PDBrestore, PDBfixer is available as a command‐line application. CHARMM‐gui is a web‐based platform, freely accessible for academic users through registration, designed to interactively build complex systems, including ligand‐protein, aimed at producing the input files for the CHARMM MD suite [41]. Like PDBrestore, CHARMM‐GUI can cope with gap restoration and generate ligands and cofactors parameterization for MD applications.

The two PDB structures examined here are indeed challenging examples, presenting many structural features that require repair or optimization. The SARS‐CoV‐2 helicase 7NNG contains two symmetry‐related protein chains, each with approximately 4,500 heavy atoms, coordinated to three zinc ions located in the zinc‐binding domain and one ligand (UJK) positioned at the nucleotide binding site [42, 43]. Chain A of 7NNG has three internal gaps, totaling 20 missing residues (95–102, 186–192, 203–207), while chain B has two internal gaps (204–207, 337–339), totaling 7 missing residues.

The human TDO inhibitor complex 6A4I [44] consists of four equivalent protein chains (each with approximately 2,600 heavy atoms) bound to the prosthetic HEME group. These chains exhibit a total of twelve internal gaps, with chains A (172–182, 244–247, 339–443), B (169–184, 239–252, 339–346), C (179–184, 243–249, 339–345), and D (169–184, 228–261, 340–347) missing 20, 38, 20, and 48 residues, respectively.

In Table 2, we summarize the results obtained using the three web applications on the 7NNG and 6A4I chains. In bold font, we report the correct number of atoms (total and added hydrogen atoms) based on the residue sequence specified in SEQRES, excluding the missing C‐ and N‐terminal residues (i.e., including only the internal gaps) and considering the protonation state of the amino acids at pH = 7. At this pH:

- Histidine is neutral,
- Arginine and lysine are positively charged,
- Glutamic acid and aspartic acid are deprotonated, and
- Cysteine is protonated unless it is involved in a disulfide bridge or coordinating a metal (as in 7NNG).

**TABLE 2:** PDBrestore, CHARMM‐gui, and PDBfixer results on 7NNG and 6A4I.

	7NNG						
	Chain	N	$N_{h}$	$N_{L}$	Gaps	UJK 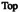	RMSD
PDBrestore	A	**9,213**	**4,593**	**23**	3	yes	0.49
PDBfixer	A	9,221	4,601	24	3	no	0.00
CHARMM‐gui	A	9,222	4,602	**23**	1	yes	0.00
PDBrestore	B	**9,219**	4,597	**23**	2	yes	0.36
PDBfixer	B	9,227	4,605	24	2	no	0.00
CHARMM‐gui	B	9,225	4,603	**23**	2	yes	0.00

	6A4I						
	Chain	N	$N_{h}$	$N_{L}$	Gaps	HEM 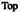	RMSD
PDBrestore	A	**5,758**	**2,873**	**73**	3	yes	0.44
PDBFIXER	A	**5,758**	**2,873**	75	3	yes	0.0
CHARMM‐gui	A	**5,758**	**2,873**	**73**	3	yes	0.00
PDBrestore	B	**5,836**	**2,911**	**73**	3	yes	0.53
PDBFIXER	B	**5,836**	**2,911**	75	3	no	0.00
CHARMM‐gui	B	**5,836**	**2,911**	**73**	2	yes	0.00
PDBrestore	C	**5,753**	**2,868**	**73**	3	yes	0.28
PDBFIXER	C	**5,753**	**2,868**	75	3	no	0.00
CHARMM‐gui	C	**5,753**	**2,868**	**73**	0	yes	0.00
PDBrestore	D	**5,858**	**2,924**	**73**	3	yes	0.37
PDBFIXER	D	**5,858**	**2,924**	75	3	no	0.44
CHARMM‐gui	D	**5,858**	**2,924**	**73**	1	yes	0.00

For both chains A and B of the 7NNG structure, PDBrestore successfully joins all gaps, matching the expected number of atoms based on the sequence for each chain. PDBrestore also correctly assigns eight deprotonated cysteines coordinating the zinc ions in the zinc‐binding domain.

PDBfixer successfully joins all gaps but incorrectly adds 8 extra hydrogen atoms for both chains A and B, corresponding to the HG hydrogen atoms in zinc‐coordinating cysteines.

CHARMM‐GUI fails to restore the gaps 95–102 and 203–204 in chain A and incorrectly protonates eight zinc‐coordinating cysteines and GLU32. In chain B of 7NNG, CHARMM‐GUI correctly joins the two gaps but identifies only three out of eight deprotonated cysteines.

Regarding the ligand, both CHARMM‐GUI and PDBrestore identify the correct protonation state at pH = 7 (with the same number of added hydrogen atoms) and provide parameterization files in a popular format that can be easily converted for common MD suites such as GROMACS [28] or AMBER [32]. However, PDBfixer adds an extra hydrogen atom to the UJK ligand, incorrectly protonating one of the oxygen atoms in the carboxylic moiety.

For the 6A4I structure, the three web applications add the same number of hydrogen atoms consistently across all four chains, irrespective of the number of gaps. However, while both PDBrestore and PDBfixer successfully restore the gaps in all four chains, CHARMM‐GUI fails in this task for chain B (gap 339–346), chain C (all three gaps are disjoined), and chain D (gap 340–347). Additionally, PDBfixer incorrectly protonates the two carboxylic groups in the HEME ligand.

As shown in Table 2, PDBrestore and PDBfixer emerge as the most reliable tools for fixing the gap‐rich 7NNG and 6A4I PDB structures. However, in PDBfixer, gap structures are optimized while keeping the rest of the coordinates fixed at their experimental values. While limiting gap optimization to missing residues or side‐chain atoms is computationally convenient, it may not be the best solution for “fixing” a defective PDB file for MD applications.

Beyond macroscopic defects such as gaps or missing side‐chain atoms, raw PDB atomic coordinates from X‐ray data are often characterized by suboptimal bond lengths, distorted bending or torsional angles, or unphysical non‐bonded contacts. These issues most frequently involve residues with polar side chains, which are more commonly found on the surfaces of globular proteins [45]. The presence of these point defects can prevent the straightforward use of “repaired” PDB structures in MD applications. Large initial potential energy gradients (evaluated using reliable force fields) due to a few suboptimal structural features may cause MD simulations to crash at the start. Identifying these structural defects can be a challenging and time‐consuming process for end‐users, requiring further intervention on the “repaired” PDB coordinates.

Unlike PDBfixer and CHARMM‐GUI, PDBrestore performs a fast conjugate gradient optimization of the entire system, including the added missing residues, cofactors, and metals, using a well‐established force field for proteins [30] (see Figure 1). This minimization process, while involving limited structural readjustment of backbone atoms (as measured by the reported RMSD in Table 2), effectively repairs subtle structural flaws related to non‐optimal internal coordinates or non‐bonded contacts. The resulting optimized PDB file is characterized by a negative total energy and a potential energy gradient modulus below the threshold of 10 kJ/mol/Å per atom.

**FIGURE 5:**
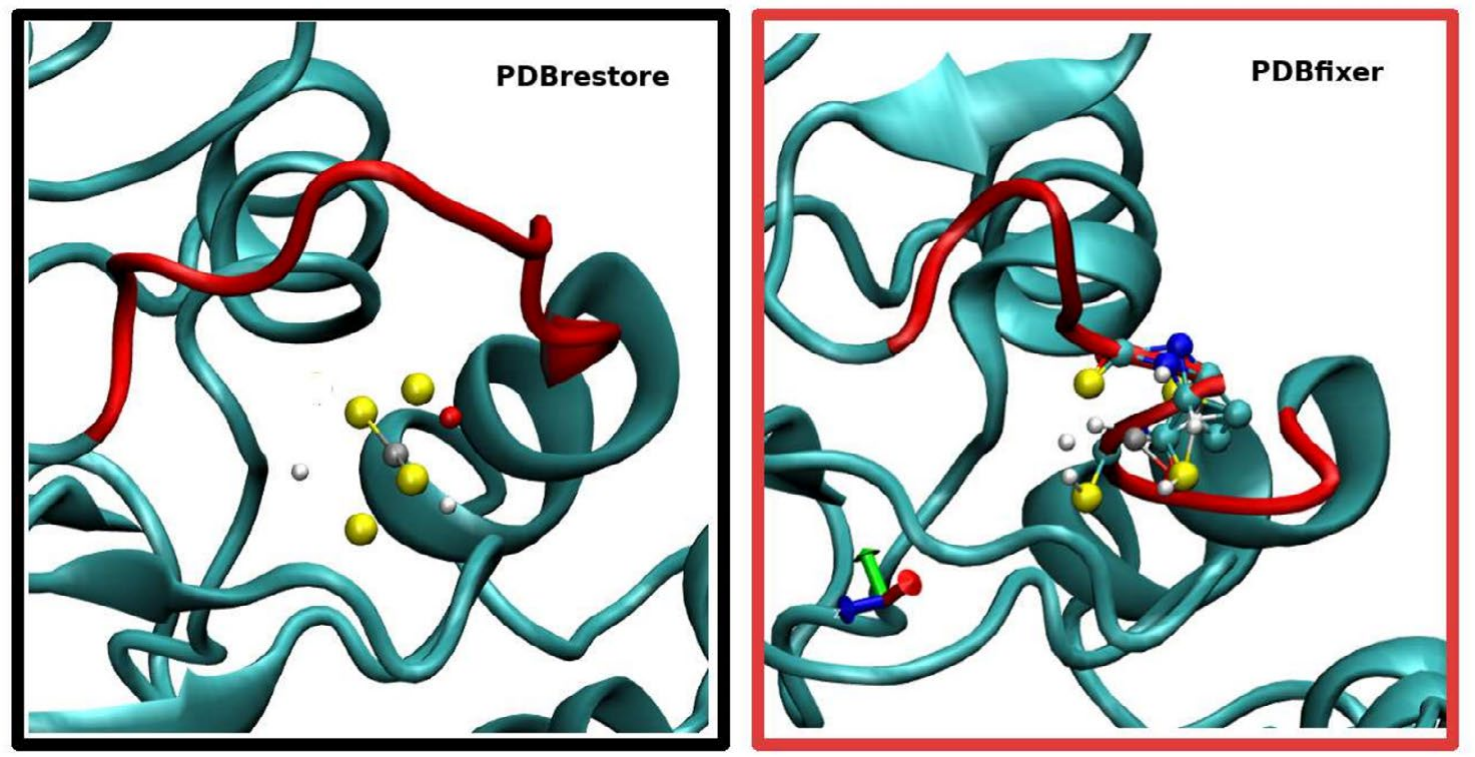
Structural details of the fixed PDB structures according to PDBrestore and PDBfixer of the Zinc domain of chain A of 7NNG. Gaps are highlighted in red. Ball‐and‐stick representation refers to atoms within 3.0 Å of the Zinc ion (in gray color).

In Figure 5, we compare a magnified image of the region of the zinc‐binding domain, including the gap 95–102 in chain A of 7NNG, after processing by PDBrestore (left) and PDBfixer (right). The domain structure is shown in a new cartoon representation, with gaps highlighted in red, while the ball‐and‐stick representation includes all atoms within 3.0 Å of the zinc atoms (shown in gray).

In the structure on the left, obtained using PDBrestore, the sulfur atoms of the four coordinating cysteines and the carboxylic oxygen of ASP101 in the gap are located within 3.0 Å of the zinc ion, with no apparent unusually close non‐bonded contacts. In contrast, the structure on the right, delivered by PDBfixer, shows several atoms in the gap 95–102 exhibiting steric clashes with the (incorrectly) protonated cysteine residues. VMD assigns bonds between non‐bonded atoms in close contact, indicating significant structural issues.

As any MD practitioner can easily guess, using the PDBfixer coordinates as a starting configuration for chain A of 7NNG—unlike the PDBrestore output—may cause the simulation to fail immediately due to excessive initial non‐bonded forces.

## 4 Conclusions and Perspectives

We have presented PDBrestore, a freely accessible web application for repairing raw PDB files downloaded from the Protein Data Bank. Through a simple HTML platform requiring minimal end‐user intervention, PDBrestore adds hydrogen atoms, adjusts metal coordination, and models missing side‐chain atoms or residues, enforces post‐translational modifications such as disulfide bridges, inserts ligands, and optionally generates a solvated water MD box for advanced MD applications. The performance of PDBrestore in repairing defective PDB files has been tested on more than 20,000 protein chains randomly extracted from the PDB, achieving a success rate of approximately 84%.

PDBrestore has been compared to two popular alternatives, PDBfixer and CHARMM‐GUI, which are also accessible via web servers, using two challenging raw PDB files: The SARS‐CoV‐2 helicase 7NNG and the TDO inhibitor complex 6A4I. For these two examples, which exhibit multiple defects such as incomplete side chains and extended gaps, we have shown that PDBrestore, unlike the other tested platforms, is capable of delivering a reasonably optimized structure ready for MD applications without requiring further end‐user intervention.

However, PDBrestore has some important limitations. In its current implementation, the end‐user can select only one protein chain and one ligand at a time, reflecting its focus on quickly providing a receptor‐ligand system ready for MD applications in high‐level drug discovery screening. While multiple chains or protein complexes with multiple ligands can be constructed by combining multiple PDBrestore outputs, this approach is impractical and time‐consuming, requiring significant end‐user intervention and editing of the output files. Additionally, PDBrestore does not currently support DNA or RNA chains and has limited capabilities in handling non‐standard amino acids, such as phosphorylated, glycosylated, or acetylated residues, or non‐amino acid components like ornithine or citrulline. Many of the interface's failures are due to these limitations.

Despite these constraints, PDBrestore is able to deliver, with a reasonably high success probability, a good starting point for a ligand‐receptor *holo* structure for MD applications aimed at computing binding free energies using fully atomistic modeling. The PDBrestore web server is currently running on a low‐end dedicated machine featuring an AMD FX‐8150 Eight‐Core Processor with 8 GB of memory. The same server also handles ORAC [29] downloads and PrimaDORAC [31] requests. Due to hardware limitations and the current server workload, end‐user requests for PDBrestore are executed individually using a simple queue‐management system. While the current hardware setup is sufficient for handling requests involving the repair of an average protein‐ligand system, it is likely inadequate for more complex tasks involving multiple protein chains or large protein complexes with multiple ligands or cofactors.

In the future, we plan to migrate the application to more powerful hardware and extend its capabilities to include support for nucleic acids, non‐standard residues, multiple ligands, and multiple protein chains in a single end‐user interaction. This upgrade will also allow for multiple requests to be processed simultaneously on the server.

## Data Availability

Data that support the findings of this study are available in ORAC, PDBrestore at http://www1.chim.unifi.it/orac. These data were derived from the following resources available in the public domain: http://www1.chim.unifi.it/orac, http://www1.chim.unifi.it/orac. Access to the PDBretore web‐interface (www1.chim.unifi.it/orac/pdbrestore) is unrestricted with no need for registration or licence obligations except those provided in the GPL license (version 1) of the ORAC suite. The PDBrestore bash wrapper is available upon downloading the open‐source ORAC distributions, freely accessible at the website www1.chim.unifi.it. Software and configuration requirements are specified in the building instructions of the ORAC suite.

## References

[1] A. V. Sadybekov and V. Katritch , “Computational Approaches Streamlining Drug Discovery,” Nature 616 (2023): 673–685. 37100941 10.1038/s41586-023-05905-z

[2] N. Deng , S. Forli , P. He , et al., “Distinguishing Binders From False Positives by Free Energy Calculations: Fragment Screening Against the Flap Site of HIV Protease,” Journal of Physical Chemistry B 119 (2015): 976–988. 25189630 10.1021/jp506376z PMC4306491

[3] L. Wang , Y. Wu , Y. Deng , et al., “Accurate and Reliable Prediction of Relative Ligand Binding Potency in Prospective Drug Discovery by Way of a Modern Free‐Energy Calculation Protocol and Force Field,” Journal of the American Chemical Society 137 (2015): 2695–2703. 25625324 10.1021/ja512751q

[4] V. Gapsys , L. Perez‐Benito , M. Aldeghi , et al., “Large Scale Relative Protein Ligand Binding Affinities Using Non‐Equilibrium Alchemy,” Chemical Science 11 (2020): 1140–1152. 10.1039/c9sc03754c PMC8145179 34084371

[5] Z. Cournia , B. K. Allen , T. Beuming , D. A. Pearlman , B. K. Radak , and W. Sherman , “Rigorous Free Energy Simulations in Virtual Screening,” Journal of Chemical Information and Modeling 60 (2020): 4153–4169. 32539386 10.1021/acs.jcim.0c00116

[6] A. Özen , E. Perola , N. Brooijmans , and J. Kim , Free Energy Methods in Drug Discovery: Current State and Future Directions, vol. 5 (American Chemical Society, 2021), 127–141.

[7] R. W. Zwanzig , “High‐Temperature Equation of State by a Perturbation Method. I. Nonpolar Gases,” Journal of Chemical Physics 22 (1954): 1420–1426.

[8] W. L. Jorgensen , J. K. Buckner , S. Boudon , and J. Tirado Rives , “Efficient Computation of Absolute Free Energies of Binding by Computer Simulations. Application to the Methane Dimer in Water,” Journal of Chemical Physics 89 (1988): 3742–3746.

[9] M. R. Shirts and D. L. Mobley , “An Introduction to Best Practices in Free Energy Calculations,” Methods in Molecular Biology 924 (2013): 271–311. 23034753 10.1007/978-1-62703-017-5_11

[10] G. Heinzelmann and M. K. Gilson , “Automation of Absolute Protein‐Ligand Binding Free Energy Calculations for Docking Refinement and Compound Evaluation,” Scientific Reports 11 (2021): 1116. 33441879 10.1038/s41598-020-80769-1 PMC7806944

[11] “The Advantages of Free Energy Perturbation Calculations,” (2022), https://www.schrodinger.com/products/fep.

[12] J. G. Kirkwood , “Statistical Mechanics of Fluid Mixtures,” Journal of Chemical Physics 3 (1935): 300–313.

[13] D. Branduardi , F. L. Gervasio , and M. Parrinello , “From A to B in Free Energy Space,” Journal of Chemical Physics 126 (2007): 054103. 17302470 10.1063/1.2432340

[14] V. Limongelli , M. Bonomi , and M. Parrinello , “Funnel Metadynamics as Accurate Binding Free‐Energy Method,” Proceedings of the National Academy of Sciences 110 (2013): 6358–6363. 10.1073/pnas.1303186110 PMC3631651 23553839

[15] G. Hummer , “Funnel Metadynamics as Accurate Binding Free‐Energy Method,” Journal of Chemical Physics 114 (2001): 7330–7337.

[16] M. Macchiagodena , M. Pagliai , M. Karrenbrock , G. Guarnieri , F. Iannone , and P. Procacci , “Virtual Double‐System Single‐Box: A Nonequilibrium Alchemical Technique for Absolute Binding Free Energy Calculations: Application to Ligands of the SARS‐CoV‐2 Main Protease,” Journal of Chemical Theory and Computation 16 (2020): 7160–7172. 33090785 10.1021/acs.jctc.0c00634 PMC8015232

[17] M. Macchiagodena , M. Karrenbrock , M. Pagliai , and P. Procacci , “Virtual Double‐System Single‐Box for Absolute Dissociation Free Energy Calculations in GROMACS,” Journal of Chemical Information and Modeling 61 (2021): 5320–5326. 34723516 10.1021/acs.jcim.1c00909 PMC8611716

[18] Schrödinger, LLC , “The AxPyMOL Molecular Graphics Plugin for Microsoft PowerPoint,” (2015), Version 1.8.

[19] A. Sali , “Comparative Protein Modeling by Satisfaction of Spatial Restraints,” Molecular Medicine Today 1 (1995): 270–277. 9415161 10.1016/s1357-4310(95)91170-7

[20] M. U. Johansson , V. Zoete , O. Michielin , and N. Guex , “Defining and Searching for Structural Motifs Using DeepView/Swiss‐PdbViewer,” BMC Bioinformatics 13 (2012): 173. 22823337 10.1186/1471-2105-13-173 PMC3436773

[21] S. Jo , T. Kim , V. G. Iyer , and W. Im , “CHARMM‐GUI: A Web‐Based Graphical User Interface for CHARMM,” Journal of Computational Chemistry 29 (2008): 1859–1865. 18351591 10.1002/jcc.20945

[22] E. L. Wu , X. Cheng , S. Jo , et al., “CHARMM‐GUI Membrane Builder Toward Realistic Biological Membrane Simulations,” Journal of Computational Chemistry 35 (2014): 1997–2004. 25130509 10.1002/jcc.23702 PMC4165794

[23] E. F. Pettersen , T. D. Goddard , C. C. Huang , et al., “UCSF Chimera–A Visualization System for Exploratory Research and Analysis,” Journal of Computational Chemistry 25 (2004): 1605–1612. 15264254 10.1002/jcc.20084

[24] J. Rodrigues , J. M. C. Teixeira , M. Trellet , and A. M. J. Bonvin , “PDB‐Tools: A Swiss Army Knife for Molecular Structures,” F1000Research 7 (2018): 1961. 30705752 10.12688/f1000research.17456.1 PMC6343223

[25] W. Humphrey , A. Dalke , and K. Schulten , “VMD–Visual Molecular Dynamics,” Journal of Molecular Graphics and Modelling 14, no. 1 (1996): 33–38. 10.1016/0263-7855(96)00018-5 8744570

[26] O. S. Nnyigide , T. O. Nnyigide , S.‐G. Lee , and K. Hyun , “Protein Repair and Analysis Server: A Web Server to Repair PDB Structures, Add Missing Heavy Atoms and Hydrogen Atoms, and Assign Secondary Structures by Amide Interactions,” Journal of Chemical Information and Modeling 62 (2022): 4232–4246. 36000562 10.1021/acs.jcim.2c00571

[27] P. Eastman , J. Swails , J. D. Chodera , et al., “OpenMM 7: Rapid Development of High Performance Algorithms for Molecular Dynamics,” PLoS Computational Biology 13 (2017): 1–17. 10.1371/journal.pcbi.1005659 PMC5549999 28746339

[28] M. J. Abraham , T. Murtola , R. Schulz , et al., “GROMACS: High Performance Molecular Simulations Through Multi‐Level Parallelism From Laptops to Supercomputers,” SoftwareX 1‐2 (2015): 19–25.

[29] P. Procacci , “Hybrid MPI/OpenMP Implementation of the ORAC Molecular Dynamics Program for Generalized Ensemble and Fast Switching Alchemical Simulations,” Journal of Chemical Information and Modeling 56 (2016): 1117–1121. 27231982 10.1021/acs.jcim.6b00151

[30] K. Lindorff‐Larsen , S. Piana , K. Palmo , et al., “Improved Side‐Chain Torsion Potentials for the Amber ff99SB Protein Force Field,” Proteins 78 (2010): 1950–1958. 20408171 10.1002/prot.22711 PMC2970904

[31] P. Procacci , “PrimaDORAC: A Free Web Interface for the Assignment of Partial Charges, Chemical Topology, and Bonded Parameters in Organic or Drug Molecules,” Journal of Chemical Information and Modeling 57 (2017): 1240–1245. 28586207 10.1021/acs.jcim.7b00145

[32] R. Salomon‐Ferrer , D. A. Case , and R. C. Walker , “An Overview of the Amber Biomolecular Simulation Package,” Wiley Interdisciplinary Reviews: Computational Molecular Science 3, no. 2 (2013): 198–210.

[33] “GAFF and GAFF2 are Public Domain Force Fields and are Part of the AmberTools Distribution,” Available for Download at https://amber.org. Internet Address (accessed January, 2022). According to the AMBER Development Team, the Improved Version of GAFF, GAFF2, is an Ongoing Project Aimed at “Reproducing Both the High Quality Interaction Energies and Key Liquid Properties Such as Density, Heat of Vaporization, and Hydration Free Energy”. GAFF2 is Expected “to be an Even More Successful General Purpose Force Field and that GAFF2‐Based Scoring Functions Will Significantly Improve the Successful Rate of Virtual Screenings.”

[34] P. Li , B. P. Roberts , D. K. Chakravorty , and K. M. J. Merz , “Chemical–Physical Analysis of a Tartrate Model Compound for TACE Inhibition,” Journal of Chemical Theory and Computation 9 (2013): 2733–2748.23914143

[35] W. L. Jorgensen , J. Chandrasekhar , J. Madura , R. Impey , and M. Klein , “Comparison of Simple Potential Functions for Simulating Liquid Water,” Journal of Chemical Physics 79, no. 2 (1983): 926–935.

[36] M. Parrinello and A. Rahman , “Crystal Structure and Pair Potentials: A Molecular‐Dynamics Study,” Physical Review Letters 45 (1980): 1196–1199.

[37] M. Marchi and P. Procacci , “Coordinates Scaling and Multiple Time Step Algorithms for Simulation of Solvated Proteins in the NPT Ensemble,” Journal of Chemical Physics 109 (1998): 5194–5202.

[38] S. Nosé , “A Unified Formulation of the Constant Temperature Molecular Dynamics Methods,” Journal of Chemical Physics 81 (1984): 511–519.

[39] S. Izadi and A. V. Onufriev , “Accuracy Limit of Rigid 3‐Point Water Models,” Journal of Chemical Physics 145 (2016): 074501. 27544113 10.1063/1.4960175 PMC4991989

[40] A. Hijikata , K. Yura , T. Noguti , and M. Go , “Revisiting Gap Locations in Amino Acid Sequence Alignments and a Proposal for a Method to Improve Them by Introducing Solvent Accessibility,” Proteins 79, no. 6 (2011): 1868–1877. 21465562 10.1002/prot.23011 PMC3110861

[41] A. D. MacKerell , D. Bashford , M. Bellott , et al., “All‐Atom Empirical Potential for Molecular Modeling and Dynamics Studies of Proteins,” Journal of Physical Chemistry 102 (1998): 3586–3616. 10.1021/jp973084f 24889800

[42] J. A. Newman , A. Douangamath , S. Yadzani , et al., “Structure, Mechanism and Crystallographic Fragment Screening of the SARS‐CoV‐2 NSP13 Helicase,” Nature Communications 12 (2021): 4848. 10.1038/s41467-021-25166-6 PMC8358061 34381037

[43] G. Di Paco , M. Macchiagodena , and P. Procacci , “Identification of Potential Inhibitors of the SARS‐CoV‐2 NSP13 Helicase via Structure‐Based Ligand Design, Molecular Docking and Nonequilibrium Alchemical Simulations,” ChemMedChem 19 (2024): e202400095. 38456332 10.1002/cmdc.202400095

[44] G. Fu , J. Wang , G. Luo , G. Wu , and K. Qian , “Crystal Structure of Human TDO Inhibitor Complex,” (2024), https://pdbj.org/mine/summary/6a4i.

[45] C. Shao , S. Bittrich , S. Wang , and S. K. Burley , “Assessing PDB Macromolecular Crystal Structure Confidence at the Individual Amino Acid Residue Level,” Structure 30 (2022): 1385–1394.e3. 36049478 10.1016/j.str.2022.08.004 PMC9547844

